# Plasma-Treated Electrospun PLGA Nanofiber Scaffold Supports Limbal Stem Cells

**DOI:** 10.3390/polym15214244

**Published:** 2023-10-27

**Authors:** Hanan Jafar, Khalid Ahmed, Rama Rayyan, Shorouq Sotari, Rula Buqain, Dema Ali, Muawyah Al Bdour, Abdalla Awidi

**Affiliations:** 1 Cell Therapy Center, The University of Jordan, Amman 11942, Jordan; hanan.jafar@ju.edu.jo (H.J.);; 2 School of Medicine, The University of Jordan, Amman 11942, Jordan; 3 Department of Ophthalmology, School of Medicine, The University of Jordan, Amman 11942, Jordan; 4 Thrombosis Homeostasis Laboratory, School of Medicine, The University of Jordan, Amman 11942, Jordan

**Keywords:** cornea, limbal stem cells, scaffold, plasma treatment, electrospinning, nanofibers, regenerative medicine

## Abstract

The corneal epithelial layer is continuously replaced by limbal stem cells. Reconstructing this layer in vitro using synthetic scaffolds is highly needed. Poly-lactic-co-glycolic acid (PLGA) is approved for human use due to its biocompatibility and biodegradability. However, PLGA is hydrophobic, preventing cell adherence to PLGA membranes. PLGA scaffolds were prepared by electrospinning on a custom-made target drum spinning at a rate of 1000 rpm with a flow rate of 0.5 mL/h and voltage at 20 kV, then treated with oxygen plasma at 30 mA using a vacuum coater. Scaffolds were characterized by SEM, mechanically by tensile testing, and thermally by DSC and TGA. In vitro degradation was measured by weight loss and pH drop. Wettability was assessed through water uptake and contact angles measurements. Human limbal stem cells (hLSCs) were isolated and seeded on the scaffolds. Cell attachment and cytotoxicity assay were evaluated on day 1 and 5 after cell seeding. SEM showed regular fiber morphology with diameters ranging between 150 nm and 950 nm. Tensile strength demonstrated similar average stress values for both plasma- and non-plasma-treated samples. Scaffolds also showed gradual degradability over a period of 7–8 weeks. Water contact angle and water absorption were significantly enhanced for plasma-treated scaffolds, indicating a favorable increase in their hydrophilicity. Scaffolds have also supported hLSCs growth and attachment with no signs of cytotoxicity. We have characterized a nanofiber electrospun plasma-treated PLGA scaffold to investigate the mechanical and biological properties and the ability to support the attachment and maintenance of hLSCs.

## 1. Introduction

The cornea is a highly transparent tissue located in the anterior segment of the eye, and it is responsible for more than 65% of its total optical power [1]. The external layer of the cornea, the corneal epithelium, is continuously replaced by stem cells located in a special niche called the limbus [2]. Chemical or thermal injury to these stem cells, termed limbal stem cell deficiency (LSCD), results in conjunctivalization, which refers to opacification and vascularization of the cornea due to conjunctival proliferation [3]. Techniques to repopulate the limbal stem cells utilize ex vivo expansion of a small number of cells isolated from a healthy donor eye, whether autologous or allogenic. Notably, the culture-expanded cells should be able to retain their “stemness” so as to be maintained after transplantation [4]. The “bio-engineered” tissue is transplanted to the affected eye loaded on a biologic membrane such as a fibrin substrate or an amniotic membrane [5]. The resulting tissue is difficult to handle, and only a few extremely specialized centers around the world are successfully implementing this technology.

Several attempts to reconstruct the corneal epithelium on artificial substrates have been reported. Synthetic scaffolds, such as those composed of poly(vinyl alcohol)-immobilized type I collagen and tissue-engineered collagen sponges, have demonstrated promising potential in supporting human corneal cell culture, both individually and in co-culture scenarios. These innovative platforms have shown capabilities ranging from enhancing keratocyte matrix production and promoting epithelial and endothelial cell migration to influencing cell behavior and morphology through co-culture interactions. As corneal tissue engineering aims to restore vision effectively, the utilization of these scaffold materials has garnered attention due to their biocompatibility, mechanical properties, and potential for fostering functional corneal epithelium [6,7,8]. Electrospun nanofibers derived from poly-ε-caprolactone (PCL) are another option being extensively studied in the literature. Remarkably, these nanofiber substrates have exhibited remarkable biocompatibility, fostering LEC attachment and promoting their prolific growth [9,10].

Another synthetic material of particular significance, poly-lactic-co-glycolic acid (PLGA), seems to be a favorite candidate among researchers due to its biocompatibility, biodegradability, and previous FDA approvals for human use [11]. Properties of PLGA rely greatly on the molar ratio in which lactic acid and glycolic acid are mixed. PLGA (75:25) is a copolymer of 75% polylactic acid (PLA) and 25% polyglycolic acid (PGA), which offers a balanced compromise between biocompatibility and degradation kinetics. Moreover, the molecular weight of PLGA can be adjusted to modify the mechanical properties and biodegradation rate of scaffolds. Lower molecular weights facilitate faster degradation, while higher molecular weights enhance mechanical strength, both of which can be tailored to meet the specific demands of human limbal stem cells attachment and maintenance [12].

PLGA is favored over alternatives like PLA and PCL due to its ability to finely control biodegradability, mechanical properties, and biocompatibility, making it ideal for biomedical applications [13]. While PLA and PCL are biodegradable and FDA-approved, their properties are less versatile and may not align as closely with the requirements of corneal tissue reconstruction. The ability of PLGA to achieve a balance between controlled biodegradation and mechanical strength is critical for maintaining the structural integrity of corneal scaffolds while ensuring gradual tissue regeneration. Well-established studies on PLGA-based scaffolds for corneal epithelial layer reconstruction further support the choice of PLGA [14,15].

However, PLGA is hydrophobic in nature [16], preventing proper and adequate adherence of cells to PLGA membranes and therefore limiting their potential in vivo use. Further, limited studies have reported on the ability of artificially constructed membranes to maintain the properties of limbal stem cells, not just sustain them in culture.

Synthetic scaffolds can be modified in various ways to augment cell adhesion and growth [17,18,19]. Plasma treatment is one of the most straightforward and efficient ways to enhance surface hydrophilicity and modify electric charge without adversely affecting the scaffold’s physical and chemical characteristics [20]. Additionally, plasma has good penetrability and would therefore migrate within the spaces of porous scaffolds, further enhancing the cells’ three-dimensional attachment and expansion [21].

In this study, we have created a PLGA nanofiber scaffold for corneal epithelium regeneration utilizing electrospinning, and we functionalized these scaffolds using either plasma or fibronectin. The scaffold was extensively characterized, and we aim to investigate the scaffold for its ability to support limbal stem cells in vitro.

## 2. Materials and Methods

### 2.1. Materials

RESOMER RG 755 S Poly(D,L-Lactic acid-co-glycolic acid)polymer, 75:25, inherent viscosity 0.50–0.70 dL/g (0.1% solution in CHCL3 at 25 °C), was purchased from EVONIK industries AG, Darmstadt, Germany. Dimethyl sulfoxide (DMSO) BioUltra, for molecular biology, ≥99.5% (GC), was purchased from SIGMA, Germany. Phosphate Buffer Saline (PBS), pH 7.4, Thermo Fisher Scientific, USA. Chloroform (CHCL3), HPLC grade was purchased from Tedia, Fairfield, OH, USA, and non-stick aluminum was purchased from Reynolds Wrap, USA. Cell culture materials were all purchased from Thermo Scientific, USA. CytoTox96 non-radioactive cytotoxicity assay kit was purchased from Promega, Madison, WI, USA.

### 2.2. Preparation

A viscous polymer solution with a concentration of 22.5% *wt*/*v* was prepared by dissolving 0.9 g (Sartorius, Germany) of PLGA in a 4 mL solution of dimethyl sulfoxide and chloroform in a 1:3 ratio, respectively. The solution was added to the PLGA gradually with regular stirring using a vortex (Maxi Mix II vortex, Thermo Scientific, USA) until visibly dissolved, followed by bath sonication (Elmasonic P 70 H, Elma, Germany) for 5 min and then placed on the hot plate (Thermo Scientific, USA) with magnetic stirring overnight. An electrospinning machine (EC-DIG, IME technologies, The Netherlands) was used to prepare the scaffold at room temperature. The prepared PLGA polymer was placed in the syringe, and fibers were collected on a custom-made target drum spinning at a rate of 1000 rpm and placed at a distance 15 cm from the spinneret tip with the flow rate set at 0.5 mL/h and the voltage at 20 kV. After the scaffold was ready, it was left in the fume hood for a few hours then transferred to the vacuum oven (Barnstead, Melrose Park, IL, USA) at 37 °C for two days to remove any traces of the organic solvent.

### 2.3. Target Drum Design

A custom-made aluminum 18 × 9 cm drum was used to collect electrospun PLGA fibers. The drum consists of three uniformly distributed thin pillars surrounding a thick central bar. It was designed and assembled by the faculty of engineering—University of Jordan.

### 2.4. Plasma Treatment

PLGA scaffolds were treated with atmospheric oxygen plasma at 30 mA using a vacuum coater (EM ACE200, Leica, Germany). The scaffold was placed inside the machine under constant atmospheric pressure. The glow discharge plasma targeted the scaffold for a total of 30 s.

### 2.5. Sterilization

Two methods of sterilization were examined to decide which would be best in terms of elimination of microbial contamination while maintaining desirable physical and chemical characteristics of the scaffold.

#### 2.5.1. Gamma (γ) Irradiation

Samples were placed in sterile falcon tubes and irradiated at either 18 or 25 kGy using BIO BEAM GM 3000 (Germany). After irradiation, DMEM-F12 media was added to the sample and incubated for 3 days at 37 °C, 5% CO_2_. The media was then collected for aerobic and anaerobic cultures, while the samples were retrieved and underwent the same battery of tests for characterization.

#### 2.5.2. Alcohol Disinfection Followed by UV Radiation

PLGA scaffolds were soaked in 70% Ethanol for 2 min, followed by UV radiation for 30 min. After irradiation, media was added as described above and checked for microbial growth. Samples were also retrieved and examined as described above.

### 2.6. Characterization

#### 2.6.1. Scanning Electron Microscopy

Samples were placed on carbon-adhesive stubs, coated with 4 nm gold using Leica EM ACE200 then imaged at 2 Kv using Versa 3D, FEI (Thermo, USA).

#### 2.6.2. Porosity

To calculate porosity (Φ), the PLGA scaffolds were cut into three 52 × 20 mm rectangular pieces and the thickness measured for each using a digital caliper (Mityutoyo, Japan). Following that, each sample was weighted, and its density was derived. Using Equation (1) and the standard density of 1.3 mg/mm^3^, porosity was calculated.

(1) $$\Phi \left(\%\right)=(1-\frac{\rho }{\rho_{s}})$$

ρ resembles density and $\rho_{s}$ resembles the standard density.

#### 2.6.3. Mechanical Strength

The PLGA scaffold’s tensile strength was measured in relation to plasma treatment at room temperature using a universal testing machine (UTM) (EZ-LX, Shimadzu, Japan). A triplicate of PLGA scaffold samples before plasma treatment was cut into 10 × 50 mm rectangles with a uniform thickness of 0.050 mm, and an identical triplicate was cut from the scaffold after plasma treatment. Each test run consisted of one sample fixed firmly in position by the machine’s top mobile and bottom non-mobile metal clamps with an average gauge length of 30 mm. The samples were then stretched upwards at a constant loading rate of 10 mm/min until complete tear. The test ran a total of six times, and results both before and after plasma exposure were collected and displayed on stress–strain curves.

#### 2.6.4. Thermal Analysis

Differential scanning calorimetry (DSC-60A plus, Shimadzu, Japan) was performed on raw PLGA, non-plasma-treated electrospun PLGA, and plasma-treated electrospun PLGA nanofibers in order to accurately determine the Tg temperature. Each sample was prepared for testing by being placed in a mini aluminum dish that was covered by a lid and finally sealed using a sample sealer. The samples were then placed in the DSC machine at a starting temperature of 30 °C then gradually heated until reaching the 200 °C mark with a constant heating rate of 10 °C/min. Following that, the samples were quickly cooled to −50 °C under the influence of liquid nitrogen and a cooling rate of 20 °C/min. The second and last heating runs took the samples back up to 200 °C at a heating rate of 10 °C/min. The second heating run results were graphed and analyzed to further study the samples’ temperature behaviors.

Thermogravimetric analysis (TGA) was also conducted for the samples by putting them in recyclable 70 μm alumina oxide crucibles (ME-24123, Mettler Toledo, Switzerland) that were then loaded onto the TA machine (TGA/DSC 2, Mettler Toledo, Switzerland) and heated until reaching 500 °C under a constant heating rate of 10 °C/min.

#### 2.6.5. In Vitro Degradation

Twelve 10 × 60 mm samples were cut from a plasma-treated PLGA scaffold, and another dozen were cut from a non-plasma-treated scaffold in preparation for the degradation test. Each sample was weighed using the analytical balance and then placed separately in a 50 mL conical tube containing 10 mL of phosphate buffer saline (PBS; pH 7.4), which was not renewed throughout the 12-week process. The tubes were placed in a microbiological incubator (Heratherm IMH60-S, Thermo Scientific, USA) under dynamic conditions by laying them on a shaker (compact digital mini rotator, Thermo Scientific, USA) set at 60 rpm and a temperature of 37 °C. Every week, a sample from each the plasma-treated and non-plasma-treated groups were taken out of the solution, rinsed, and left to dry on a filter paper before being weighed again, and PH levels of PBS were monitored by a PH meter (Orion 266S, Thermo electron corporation, Germany). Readings were compared to their initial values and plotted.

#### 2.6.6. Wettability

In order to assess the PLGA scaffold’s wettability, water uptake readings were recorded and contact angles of water droplets on its surface were measured.

For water uptake measurements, samples were cut from both non-plasma- and plasma-treated PLGA scaffolds and had their initial masses measured. Samples were then inserted in 15 mL conical tubes and completely covered by de-ionized water. Afterwards, the samples were placed in the incubator under static conditions for 24 h, through which samples were taken out at the 2 and 24 h marks and left for 5 min on filter paper to dry and weighed again. Water uptake was then calculated using the wet mass and initial mass according to equation [22].

(2) $$water\;uptake\left(\%\right)=\frac{m_{1}-m_{0}}{m_{0}}\times 100$$

$m_{0}$ resembles the initial mass, whilst $m_{1}$ resembles the final mass

For contact angle measurements, a 10 × 10 mm sample was cut off from the PLGA scaffold before the plasma treatment and another identical one after the plasma treatment. The two scaffolds were fixed in place on a petri dish surface using double-faced tape. A USB digital microscope was set up and connected to a laptop. Four pictures of four different droplets on each scaffold sample were taken within 10 s of contact with 3 µL de-ionized water droplets. Each contact angle was measured on the ImageJ 1.53a image processing software using the “Drop analysis—LB-ADSA” plugin, and the results were averaged for both plasma-treated and non-plasma-treated samples.

### 2.7. Biological Testing

#### 2.7.1. Limbal Stem Cells Isolation

Limbal biopsies were obtained from healthy donors (n = 3) undergoing other procedures. Informed consents were obtained from patients for sample collection. All samples were processed within 3–6 h of collection procedure. Limbal stem cells (LSCs) were isolated from corneal biopsy tissue samples as described previously [23]. Briefly, samples were washed thoroughly in PBS and trypsinizatied (0.05% Trypsin-EDTA) for 80 min at 37 °C under contentious agitation (100 rpm). Cells were collected every 20 min. Cells were cultured (1.5 × 10^4^/cm^2^) onto a feeder layer of lethally irradiated 3T3-J2 cells in complete medium: DMEM and Ham’s F12 media (2:1 mixture) containing 10% FCS, adenine (0.18 mM), insulin (5 μg/mL), cholera toxin (0.1 nM), triiodothyronine (2 nM), hydrocortisone (0.4 μg/mL), glutamine (4 mM), and penicillin–streptomycin (50 IU/mL). Epidermal growth factor (10 ng/mL) was added starting at day 3. Subconfluent primary cultures were passaged at a density of 6 × 10^3^ cells/cm^2^ and cultured as above. All cultures were incubated at 37 °C in a 5% CO_2_ atmosphere. For experiments involving cultures on PLGA scaffolds, PLGA scaffolds treated with fibronectin were used as positive control.

#### 2.7.2. Cell Attachment

LSCs (6 × 10^4^ cells/well) were co-cultured with 3T3-J2 irradiated cells for 5 days in: (1) tissue culture 6-well plate (control), (2) PLGA treated with fibronectin, and (3) PLGA treated with plasma. For fibronectin treatment, fibronectin was coated onto plates at a concentration of 0.01 mg/mL for 2 h at 37 °C, followed by a 1 h air-drying step at room temperature. At day 1 and day 5, cells were washed with PBS and fixed in 4% formaldehyde for 15 min. Cells were then stained with ProLong™ Diamond Antifade Mountant with DAPI. Stained cells were observed under fluorescent microscope.

#### 2.7.3. Cytotoxicity Assay

Limbal stem cells were cultured on: (i) PLGA treated with fibronectin, (ii) PLGA treated with plasma, and (iii) tissue culture treated plates as a control. After incubation at 37 °C for 72 h, supernatant was collected to detect the lactate dehydrogenase (LDH) release with a CytoTox96 non-radioactive cytotoxicity assay kit according to the manufacturer’s instruction. Finally, the OD was measured at 490 nm in the microplate spectrophotometer (GloMax, Promega, USA).

### 2.8. Statistical Analysis

Statistical analysis was performed using Student’s *t*-test using SPSS version 23 software. All data were expressed as mean ± standard deviation (n = 3). In all statistical evaluations, *p* < 0.05 was considered statistically significant.

## 3. Results

### 3.1. Sterilization

The gamma-irradiated samples failed to stop bacterial contamination and had a faster degradation rate than the alcohol + UV light group in our pilot study, so they were ultimately discarded from this study and subsequent testing. All results described below are on alcohol disinfected and UV radiated samples as described in the methods section.

### 3.2. Characterization

#### 3.2.1. Mechanical Strength

**Figure 1:**
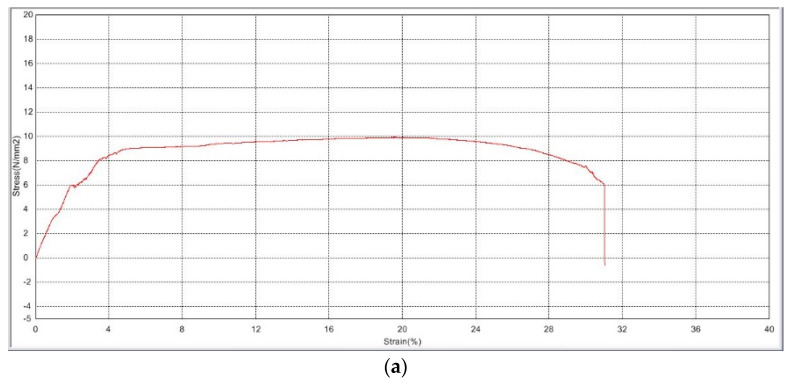

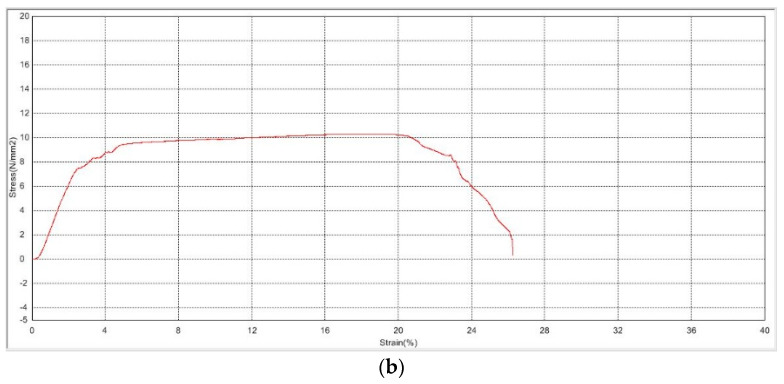
Stress–strain curves of PLGA scaffolds (**a**) before plasma treatment and (**b**) after plasma treatment.

Ultimate tensile strength (UTS) is demonstrated by the stress–strain curves for the plasma-treated and non-plasma-treated samples shown in Figure 1. The average stress the non-plasma samples were able to withstand before tear is 10.566 ± 1.16 N/mm^2^, in comparison with an average stress of 10.066 ± 1.00 N/mm^2^ for the plasma-treated samples. Statistical analysis does not show a significant difference between the values (*p* = 0.6), indicating a very minimal change in mechanical properties and elasticity, which suits the aim of the study.

#### 3.2.2. Thermal Analysis

- DSC

**Figure 2:**
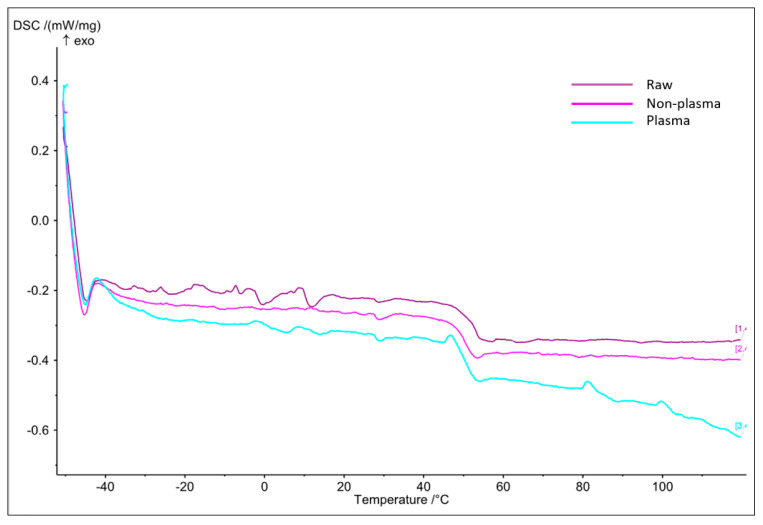
DSC graph for the raw, non-plasma-treated, and plasma-treated samples.

Differential scanning calorimetry (DSC) is an invaluable procedure in studying a sample’s glass transition behaviors in relation to increasing temperatures. Figure 2 illustrates three graphs for each of the raw, electrospun plasma-treated and electrospun non-plasma-treated PLGA scaffolds. We can observe that the electrospun plasma-treated PLGA sample had very similar glass transition (Tg) temperature (50.1 °C), as compared to 51.5 °C and 51.3 °C for the raw and electrospun non-plasma-treated scaffolds, respectively. This suggests that plasma treatment has a negligible effect on the resistance of samples to thermal behavior, which is favorable in cell culture applications.

b. TGA

**Figure 3:**
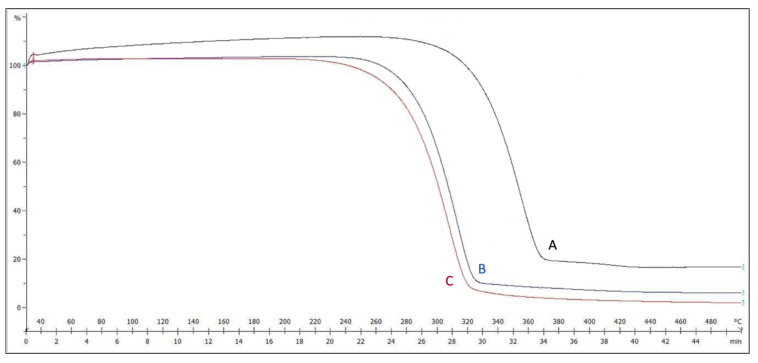
TGA graph for the (A) non-plasma-treated, (B) plasma-treated, and (C) raw samples.

Thermal gravimetric analysis is used to accurately specify the degradation (weight loss temperature) of a given material and, thus, how much heat it can withstand. Figure 3 shows the TGA curves for the weighted samples. Weight loss starts at around (277 °C) for the non-plasma-treated electrospun sample, as opposed to (230 °C) for the plasma-treated one. Such a big difference confidently confirms the effect of plasma treatment on reducing the degradation point of the PLGA samples, which is more favorable in cell culture applications.

#### 3.2.3. Degradation

In vitro degradation was estimated by studying the morphology, weight loss, and PH changes for the samples. According to previous research, a relationship between plasma treatment and biodegradability of electrospun PLGA scaffolds has been established [24,25].

- Morphology

**Figure 4:**
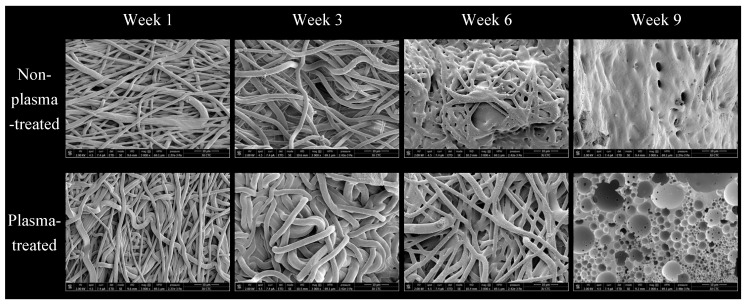
Scanning electron microscopy (SEM) micrographs of non-plasma-treated nanofibrous scaffolds and plasma-treated nanofibrous scaffolds at a magnification of ×3000 at weeks 1, 3, 6, and 9.

Figure 4 shows SEM pictures of plasma- and non-plasma-treated samples taken at different week intervals throughout the test. They show gradual degradation and weight loss over time. Notice that, after approaching week ten, the samples became so small and shrunk to the point where they could not be handled and managed.

b. Weight Loss

**Figure 5:**
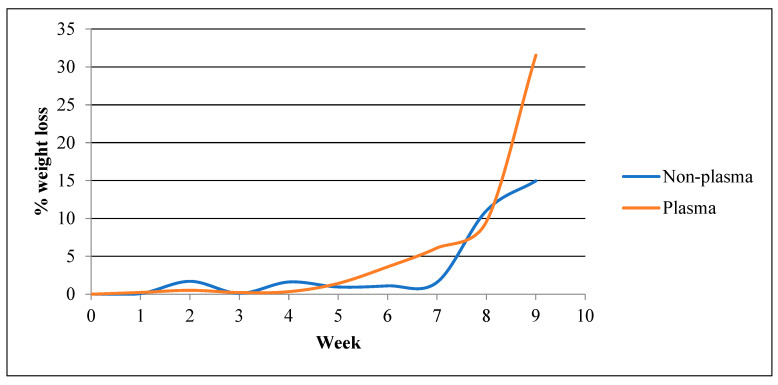
Weight loss over time for the plasma-treated vs. non-plasma-treated samples.

Figure 5 compares weight loss progress of plasma-treated samples as opposed to non-plasma-treated samples over the 84-day course. Weeks one to seven witnessed extremely negligible degradation regarding the non-plasma-treated samples, with the average weight loss being 1.02% ± 0.68. After the eighth week, however, the sample showed a remarkable fall in mass (11.03%) followed by a 14.97% mass loss after the ninth week. Once the tenth week was reached, the samples were too decomposed to be handled and weighed, indicating a complete degradation. On the other hand, the plasma-treated samples started showing somewhat noticeable mass loss at a slightly faster rate, specifically after the sixth (3.61%) and seventh (6.11%) weeks of dynamic incubation. Surprisingly, the eighth week showed less degradation compared to the non-plasma-treated, 9.59% and 11.03%, respectively. But quickly after, it showed massive degradation after the ninth week (31.56%), displaying the clearest contrast between the two groups. Similarly to the non-plasma group, the samples were too dissolved to be handled from week ten and beyond.

c. pH changes

**Figure 6:**
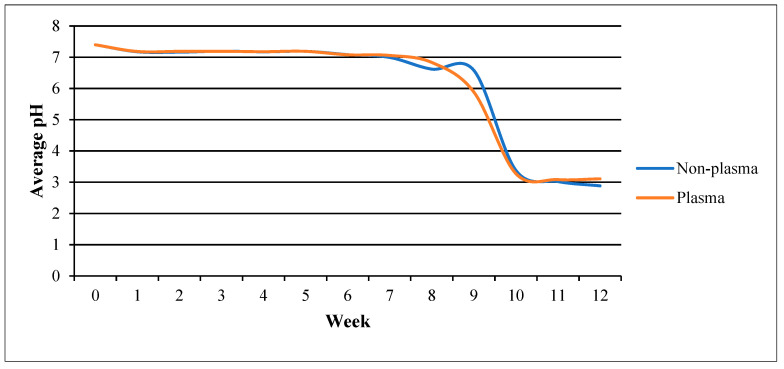
pH changes over time for the plasma-treated vs. non-plasma-treated samples.

Figure 6 compares the pH change between the plasma-treated and the non-plasma-treated samples. Both samples showed a similar pattern of negligible decrease from the starting pH (7.4) up to the seventh week. The non-plasma-treated samples showed a slight bump before both groups sharply increased in acidity and intersected at week 11 (pH 3.0). Subsequently, both groups reached a plateau with the non-plasma-treated scaffold being slightly more acidic, with a difference of 0.225. Increase in acidity indicates degradation of the PLGA scaffold, as it is due to the accumulation of lactic and glycolic acid [26].

#### 3.2.4. Wettability

The wettability of the PLGA scaffolds was assessed via the water uptake measurements and the contact angle values of water droplets on its surface.

- Water Uptake

The scaffold’s absorptive potential is a powerful tool in studying its water behavior. The triplicate of non-plasma-treated samples was compared to its plasma-treated counterparts. Table 1 shows the intervals at which samples were taken out and their average water uptake percentage calculated. The results showed minimal decrease in mass among the non-plasma-treated group and very minimal increase in mass among the plasma-treated samples. This relative hydrophilicity at each time interval is appropriate for corneal limbal stem cell cultures, although it failed to demonstrate statistically significant results.

b. Contact Angle

**Figure 7:**
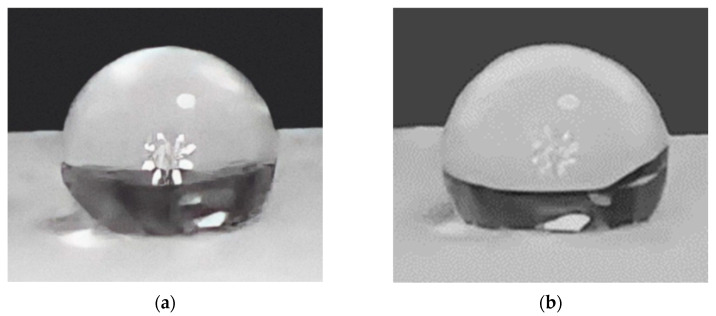
(**a**) Water droplet on the scaffold before the plasma treatment; angle of 142.0°. (**b**) Water droplet on the scaffold after the plasma treatment; angle of 132.4°. This illustrates the increase in water absorption after plasma treatment.

Contact angle is a powerful indicator of PLGA scaffolds’ hydrophilicity. Table 2 shows the contact angles before and after the plasma treatment. The contact angle (Figure 7) between the de-ionized water droplets and the non-plasma treated PLGA samples averaged about 143.2 ± 1°, while the plasma treated samples’ contact angles averaged at around 130.9 ± 1.4°. The hydrophilicity of the scaffolds is inversely related to the contact angle [22]; in this case, the average contact angle value decreased by 12.3°, which indicates a remarkable increase in hydrophilicity after plasma treatment [27]. Statistical analysis shows an extremely significant difference between the contact angles of both samples (*p* < 0.05). Therefore, it may be more suitable for limbal stem cell adhesion, proliferation, and growth [12].

### 3.3. Scanning Electron Microscopy

**Figure 8:**
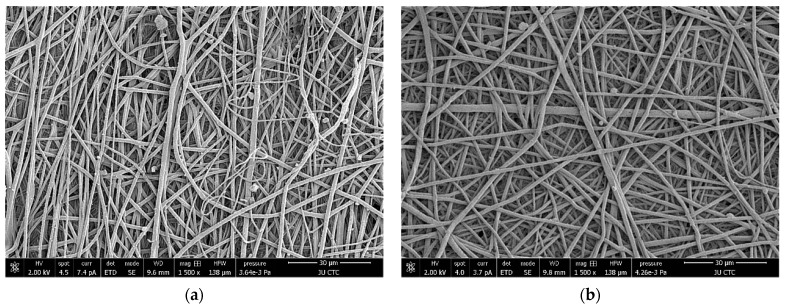
(**a**) Scanning electron microscopy (SEM) micrograph of raw non-plasma-treated nanofibrous scaffold and (**b**) raw plasma-treated nanofibrous scaffold, both captured at a magnification of ×1500.

Figure 8a,b show the morphology of the randomly aligned fibers of raw plasma-treated and non-plasma-treated PLGA scaffolds, respectively. The average PLGA fiber diameter was calculated by averaging manually taken readings. The non-plasma fiber diameter was 822 nm with a standard deviation of 175 nm, whilst the plasma fiber diameter was 1.367 μm with a standard deviation of 374 nm. This demonstrates a noticeable and statistically significant increase (*p* < 0.05). While the porosity of the nanofiber-based non-plasma-treated 75:25 PLGA scaffolds was calculated to be 81.52%.

### 3.4. Biological Testing

Prior to commencing biological testing, it was essential to confirm sterility of the scaffold. Both aerobic and anaerobic cultures were negative following incubation of the scaffolds that were sterilized using the methods described in the methodology. The authors opted to use gamma irradiation sterilization over alcohol and UV disinfection to facilitate future preclinical testing.

#### 3.4.1. Cell Attachment

**Figure 9:**
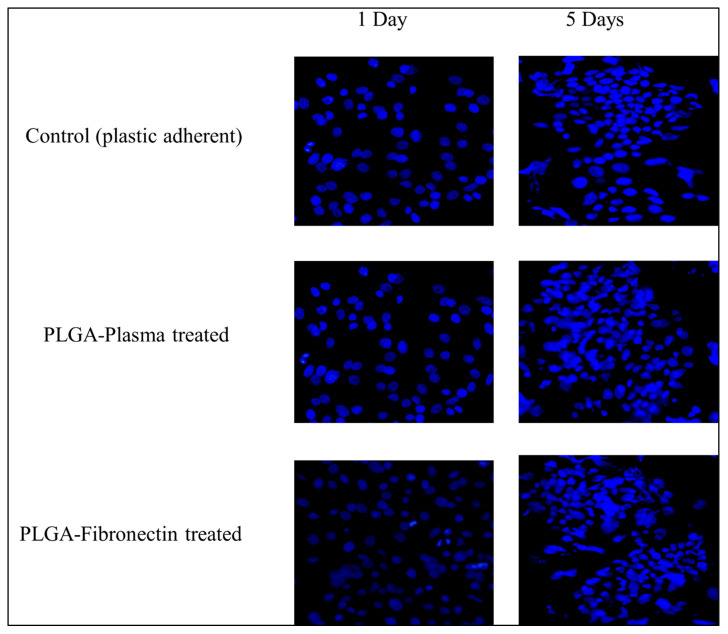
Fluorescent microscopy images of the attachment of hLSCs at days 1 and 5 in different culture conditions.

Cell attachment was analyzed for the three different attachment methods, and cell culture plastic was used as a positive control since the cells are known to adhere and spread on this material. At day 1, both the PLGA treated with plasma and fibronectin gave the same cell coverage as the control. By day 5, the LSCs grew in the form of colonies of tightly packed cells in all culture conditions (Figure 9).

#### 3.4.2. Cytotoxic Assay

**Figure 10:**
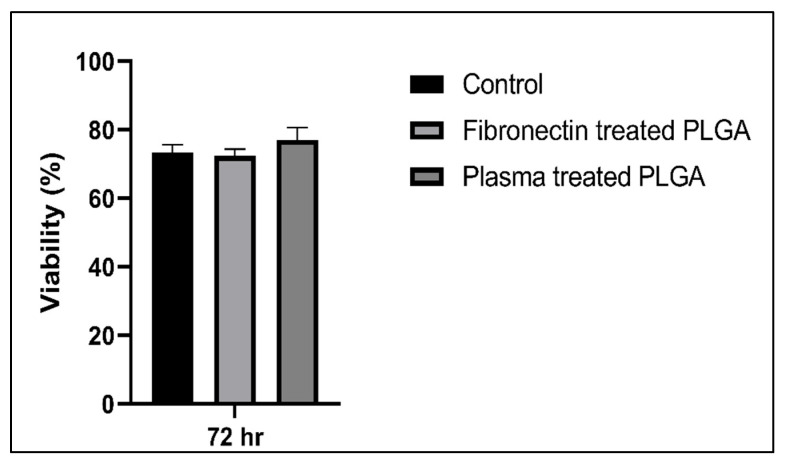
hLSCs viability graph under different culture conditions.

The biocompatibility assay showed that both fibronectin and plasma-treated PLGA scaffolds were not cytotoxic to LSCs, as the viability of cells cultured in the scaffolds appeared nearly identical to that seen in samples of cells grown in 2D culture (Figure 10).

## 4. Discussion

Over 6 million individuals worldwide suffer from blindness or severe vision impairment caused by corneal diseases, and with a worldwide donated corneal heterograft ratio of 1 cornea available for each 70 needed, a dire need for the development of an artificial corneal is evident [28,29].

The use of electrospinning of polymer fibers and fiber functionalization has enabled the fabrication of nanofibrous scaffolds that aim to be ideal for use in corneal scaffolds. Poly-lactic-co-glycolic acid (PLGA) is biocompatible, biodegradable, and FDA approved for human use. However, its hydrophobic properties prevent the proper adherence of cells to the PLGA membrane and the adequate properties of limbal stem cells.

To our knowledge, no prior studies examined PLGA nanofibers as a medium for limbal stem cell cultures. Very few studies on synthetic scaffolds for the cornea, including animal studies, have been conducted [15]. In this study, we aimed to overcome the hydrophobic properties of PLGA to enable the adherence of limbal stem cells and maintain the physiologic properties of the corneal epithelium.

Morphologically, we can not observe much difference between SEM images of samples from both groups after one and three weeks (Figure 4). Starting from the sixth week onwards, however, a stark difference in fiber morphology appears, where plasma-treated samples clearly withhold fiber shape and demarcation better than the raw samples, which start to show notable fusion. Even at week nine, after complete fusion of all fibers, we can see a much more porous morphology of the plasma-treated samples. We chose not to use gamma radiation for polymer sterilization because it is known to induce molecular weight reduction in polymers, increasing their degradation [30]. Additionally, our decision was influenced by the observed bacterial contamination in the pilot trial we conducted; data from this trial are unavailable.

Our findings demonstrate that the mechanical properties and elasticity of the PLGA scaffolds were not adversely affected by plasma treatment (Figure 1), as evidenced by the stress–strain curves for the plasma-treated and non-plasma-treated samples. This is an important finding, given the relative mechanical fragility of HAM, which is the most widely used substrate for ocular regeneration [31,32,33]. Furthermore, the electrospun plasma-treated PLGA sample had the highest glass transition (Tg) temperature (Figure 3), suggesting the beneficial role of plasma treatment in making the sample more resistant to changes in thermal behavior, which is favorable in cell culture applications. Plasma treatment also reduced thermal degradation, as demonstrated by thermal gravimetric analysis (TGA). This observation could be due to the addition of oxygen-containing functional groups to the surface of the scaffold through plasma treatment, which compromises some of the thermal integrity.

The scaffolds also showed stable in vitro degradability rates across morphology, weight, and PH parameters (Figure 4, Figure 5 and Figure 6), with significant changes beginning to manifest at the 7th and 8th weeks, respectively, for the control samples, while plasma-treated samples displayed a very similar rate. The large difference observed from that point onwards can be attributed to the effects of plasma treatment on the surface properties of the scaffolds. Plasma treatment enhances the hydrophilicity of the PLGA scaffolds, which can increase their susceptibility to hydrolytic degradation in an aqueous environment. The introduction of polar functional groups on the scaffold’s surface due to plasma treatment likely accelerates the degradation process compared to non-plasma-treated scaffolds, where the surface is less hydrophilic. This important finding is consistent for the intended surgical application for the scaffolds, as minimal circumstantial delays in operating would not see the samples decompose [24,25].

Contact angle measurements indicate a statistically significant increase in hydrophilicity after plasma treatment (Table 2) (Figure 7), which is more suitable for limbal stem cell adhesion, proliferation, and growth [12,27]. Scanning electron microscopy (SEM) showed a noticeable increase in the diameter of the plasma treated fibers (Figure 8a,b), and cell attachment and growth of LSC to the PLG, plasma-treated on day 5, grew in the form of colonies of tightly packed cells in all culture conditions, indicating an adequate property of the scaffold.

Biocompatibility assays showed that both fibronectin-treated and plasma-treated PLGA scaffolds were not cytotoxic to LSCs (Figure 9), with the viability of cells cultured in the scaffolds appearing nearly identical to that seen in samples of cells grown in 2D culture (Figure 10). This finding overcomes a major obstacle Sharma et al. faced in a similar study using PCL fibers, where the limbal epithelial cells were more metabolically active in 2D settings as compared to the surface of PCL fibers, which could hinder appropriate cell adherence [9].

## 5. Conclusions

In conclusion, our work demonstrates that plasma treatment of PLGA is an effective method to generate a working model suitable for testing in animal studies and future human studies. The scaffold shows promise as a safe and appropriate scaffold for LSC attachment and growth while demonstrating many properties that could make it a potential scaffold for future clinical application. However, further studies are needed to investigate the chemical composition and composition of the functional groups of the studied scaffolds, including their surface properties, as well as further biological testing beyond toxicity and attachment.

## Figures and Tables

**Table 1:** Water uptake in relation to plasma treatment at different time intervals. ↓ indicates a decrease in the uptake percentage, whilst ↑ indicates an increase in the uptake percentage.

Sample	2 h	24 h
Plasma average (%)	0.6 ± 0.07 ↑	0.10 ± 0.03 ↑
Non-plasma average (%)	2.71 ± 0.12 ↓	4.0 ± 0.15 ↓
*p* value	0.32	0.16

**Table 2:** Contact angles in relation to plasma treatment.

Scaffold	Average Angle
Before plasma treatment	143.2 ± 1.0°
After plasma treatment	130.9 ± 1.4°

## Data Availability

Data available upon reasonable request.
